# Bis[*N*-(pyridin-2-ylcarbon­yl)pyridine-2-carboxamidato]iron(III) perchlorate monohydrate

**DOI:** 10.1107/S1600536811047684

**Published:** 2011-11-16

**Authors:** Xi-Feng Li, Tian-Bao Qiu, Li-Xin Hu, Chun-Yue Hu

**Affiliations:** a Pharmacy College, Henan University of Traditional Chinese Medicine, Zhengzhou 450008, People’s Republic of China

## Abstract

The structure of the title salt complex, [Fe(C_12_H_8_N_3_O_2_)_2_]ClO_4_·H_2_O, contains one Fe^III^ cation, two *N*-(pyridin-2-ylcarbon­yl)pyridine-2-carboxamidate (bpca^−^) anions, one perchlorate anion and one water mol­ecule. The Fe^III^ cation has an approximate octa­hedral geometry, defined by six N atoms from two bpca^−^ anions. The nearly parallel [dihedral angle = 1.50 (1)°] bpca^−^ anions form two-dimensional supermolecules along the *a* axis by the way of weak π–π stacking inteactirons [centroid–centroid distances = 3.948 (2), 4.000 (2), 3.948 (2), 3.911 (2), 3.897 (2), 3.984 (2) and 3.929 (2) Å]. Intra- and inter­molecular C—H⋯O hydrogen bonding occurs. The water mol­ecule [occupancies 0.520 (5) and 0.480 (5)], two carbonyl O atoms [occupancies 0.622 (7) and 0.378 (7)] and the four perchlorate O atoms [occupancies 0.887 (4) and 0.113 (4)] are each disordered over two positions.

## Related literature

For the use of organic ligands containing nitro­gen hetero­atoms in the synthesis of transition-metal complexes, see: Feng *et al.* (2006 ▶); Wu *et al.* (2009 ▶); Xie & Huang (2011 ▶); Yu *et al.* (2010 ▶). For the N-donor tridentate rigid ligand Hbpca, see: Casellas *et al.* (2005 ▶); Kajiwara *et al.* (2002 ▶). For mononuclear complexes of the tridentate ligand bpca^−^, see: Madariaga *et al.* (1991 ▶); Marcos *et al.* (1989 ▶, 1990 ▶); Wocadlo & Massa (1993 ▶).
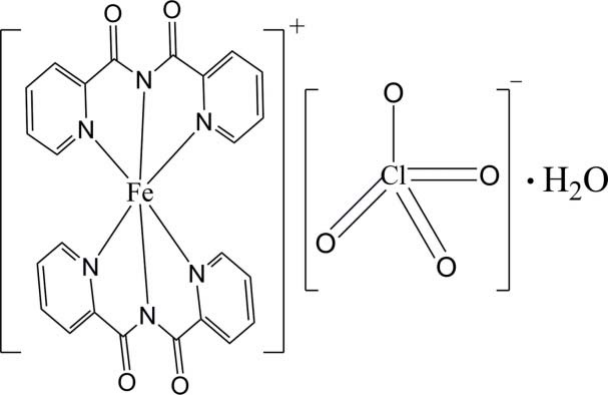


## Experimental

### 

#### Crystal data

[Fe(C_12_H_8_N_3_O_2_)_2_]ClO_4_·H_2_O
*M* *_r_* = 625.74
Triclinic, 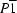

*a* = 8.8828 (8) Å
*b* = 11.7228 (3) Å
*c* = 14.4551 (9) Å
α = 109.931 (3)°
β = 103.585 (4)°
γ = 99.456 (3)°
*V* = 1325.39 (15) Å^3^
*Z* = 2
Mo *K*α radiation
μ = 0.73 mm^−1^
*T* = 298 K
0.20 × 0.14 × 0.10 mm

#### Data collection

Bruker APEXII CCD area-detector diffractometer
Absorption correction: multi-scan (*SADABS*; Bruker, 2007 ▶) *T*_min_ = 0.867, *T*_max_ = 0.930
8065 measured reflections
5880 independent reflections
4516 reflections with *I* > 2σ(*I*)
*R*_int_ = 0.021

#### Refinement

*R*[*F* ^2^ > 2σ(*F* ^2^)] = 0.053
*wR*(*F* ^2^) = 0.165
*S* = 1.06
5880 reflections
407 parameters
50 restraints
H atoms treated by a mixture of independent and constrained refinement
Δρ_max_ = 0.70 e Å^−3^
Δρ_min_ = −0.40 e Å^−3^

### 

Data collection: *APEX2* (Bruker, 2007 ▶); cell refinement: *SAINT* (Bruker, 2007 ▶); data reduction: *SAINT*; program(s) used to solve structure: *SHELXS97* (Sheldrick, 2008 ▶); program(s) used to refine structure: *SHELXS97* (Sheldrick, 2008 ▶); molecular graphics: *SHELXTL* (Sheldrick, 2008 ▶); software used to prepare material for publication: *publCIF* (Westrip, 2010 ▶).

## Supplementary Material

Crystal structure: contains datablock(s) global, I. DOI: 10.1107/S1600536811047684/jj2105sup1.cif

Structure factors: contains datablock(s) I. DOI: 10.1107/S1600536811047684/jj2105Isup2.hkl

Additional supplementary materials:  crystallographic information; 3D view; checkCIF report

## Figures and Tables

**Table 1:** Selected bond lengths (Å)

Fe1—N5	1.902 (2)
Fe1—N2	1.914 (2)
Fe1—N4	1.964 (2)
Fe1—N3	1.974 (2)
Fe1—N6	1.976 (2)
Fe1—N1	1.979 (2)

**Table 2:** Hydrogen-bond geometry (Å, °)

*D*—H⋯*A*	*D*—H	H⋯*A*	*D*⋯*A*	*D*—H⋯*A*
C12—H12⋯O3^i^	0.93	2.30	3.084 (3)	142
C12—H12⋯O4^i^	0.93	2.55	3.288 (4)	136
C2—H2⋯O5	0.93	2.52	3.292 (5)	140
C2—H2⋯O5*A*	0.93	2.41	3.116 (8)	133
C11—H11⋯O6^i^	0.93	2.62	3.385 (6)	140
C11—H11⋯O8*A*^i^	0.93	2.32	3.191 (11)	156
C16—H16⋯O6*A*^ii^	0.93	2.58	3.431 (10)	153

Symmetry codes: (i) 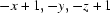; (ii) 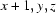.

**Fig. 1.**
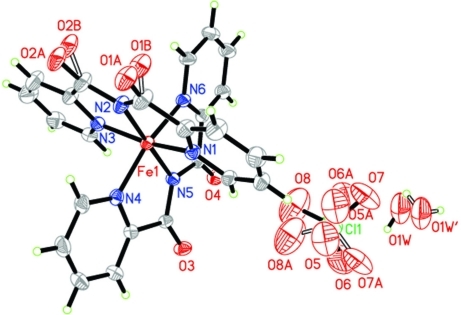
The molecular structure for title salt with isplacement ellipsoids at the 30% probability level. OW1 and OW1' atoms were disordered over two positions with approximate part occupancies of 0.520 (5) and 0.480 (5). Hydrogen atoms on OW1 and OW1' were also treated using the above two part model. O1A and O1B, were disordered over two positions with approximate part occupancies of 0.622 (7) and 0.378 (7). O5, O6, O7 and O8 were disordered with approximate part occupancies of 0.887 (4) and 0.113 (4).

## supplementary crystallographic information

### Comment

Organic ligands containing nitrogen heteroatoms play an important role in the assembling process of transition-metal complexes (Wu *et al.*, 2009; Xie *et al.*, 2011; Feng *et al.*, 2006; Yu *et al.*, 2010). The N-donor tridentate rigid ligand Hbpca (Hbpca = bis(2-pyridylcarbonyl)amine) has been attracting widespread attention (Kajiwara *et al.*, 2002; Casellas *et al.*, 2005). Mononuclear complexes of the tridentate ligand bpca^-^, [*M*(bpca)]^+^ (*M* = divalent metal ions] exemplify these types of complexed ligands (Marcos *et al.*, 1989, 1990); Madariaga *et al.*, 1991; Wocadlo *et al.*, 1993).

As a continuation of our studies related to the behaviour of the bpca ligand with Fe^III^ ions, we have synthesized the title salt complex [Fe(bpca)_2_(ClO_4_) . H_2_O]. As shown in Fig. 1, each asymmetric unit contains one Fe^III^ cation, two bpca^-^ anions, one perchlorate anion and one lattice water molecule. The bpca ligands are nearly parallel in the structure and form two-dimensional supermolecules by the way of weak π–π stacking inteactions [centroid to centroid distances = 3.9475Å, 3.9997Å, 3.9488Å, 3.9107Å, 3.8973Å, 3.9838Å and 3.9287Å, respectively].  The bond lengths for Fe—N vary from 1.902 (2)Å to 1.979 (2)Å, and the angles for N—Fe—N are between 81.65 (9)° and 178.54 (10)°. These bond lengths and angles are in agreement with those found in similar related Fe^III^ compounds (Wocadlo *et al.*, 1993).

### Experimental

A mixture of methanol and water (1:1, 2 ml) was gently layered on the top of a solution of Fe(ClO_4_)_3_.6H_2_O (46.2 mg, 0.1 mmol) in water (3 ml). A solution of Hbpca = bis(2-pyridylcarbonyl)amine (22.7 mg, 0.1 mmol), in methanol (10 ml) was added carefully as the third layer. Red crystals suitable for X-ray diffraction were obtained after 3 weeks, washed with ethanol and ether, and dried in air.

### Refinement

Lattice water OW1 and OW1' atoms were disordered over two positions with approximate part occupancies of 0.520 (5) and 0.480 (5). Hydrogen atoms on OW1 and OW1' were also treated using the above two part model. The carbonyl oxygen atoms, O1A and O1B, were disordered over two positions with approximate part occupancies of 0.622 (7) and 0.378 (7).  The four perchlorate oxygen atoms O5, O6, O7 and O8 were also disordered with approximate part occupancies of 0.887 (4) and 0.113 (4). All of the remaining H atoms were placed in calculated positions and then refined using the riding model with Atom—H lengths of 0.93Å (CH). Isotropic displacement parameters for these atoms were set to 1.2 (CH) times *U*_eq_ of the parent atom.

### Crystal data

**Table:**

[Fe(C_12_H_8_N_3_O_2_)_2_]ClO_4_·H_2_O	*Z* = 2
*M**_r_* = 625.74	*F*(000) = 638
Triclinic, *P*1	*D*_x_ = 1.568 Mg m^−^^3^
Hall symbol: -P 1	Mo *K*α radiation, λ = 0.71073 Å
*a* = 8.8828 (8) Å	Cell parameters from 3005 reflections
*b* = 11.7228 (3) Å	θ = 2.7–23.6°
*c* = 14.4551 (9) Å	µ = 0.73 mm^−^^1^
α = 109.931 (3)°	*T* = 298 K
β = 103.585 (4)°	Block, red
γ = 99.456 (3)°	0.20 × 0.14 × 0.10 mm
*V* = 1325.39 (15) Å^3^	

### Data collection

**Table:**

Bruker APEXII CCD area-detector diffractometer	5880 independent reflections
Radiation source: fine-focus sealed tube	4516 reflections with *I* > 2σ(*I*)
graphite	*R*_int_ = 0.021
phi and ω scans	θ_max_ = 27.6°, θ_min_ = 1.6°
Absorption correction: multi-scan (*SADABS*; Sheldrick, 2008)	*h* = −9→11
*T*_min_ = 0.867, *T*_max_ = 0.930	*k* = −15→10
8065 measured reflections	*l* = −18→18

### Refinement

**Table:**

Refinement on *F*^2^	Primary atom site location: structure-invariant direct methods
Least-squares matrix: full	Secondary atom site location: difference Fourier map
*R*[*F*^2^ > 2σ(*F*^2^)] = 0.053	Hydrogen site location: inferred from neighbouring sites
*wR*(*F*^2^) = 0.165	H atoms treated by a mixture of independent and constrained refinement
*S* = 1.06	*w* = 1/[σ^2^(*F*_o_^2^) + (0.0989*P*)^2^ + 0.1594*P*] where *P* = (*F*_o_^2^ + 2*F*_c_^2^)/3
5880 reflections	(Δ/σ)_max_ = 0.005
407 parameters	Δρ_max_ = 0.70 e Å^−^^3^
50 restraints	Δρ_min_ = −0.40 e Å^−^^3^

### Special details

**Table:**

Geometry. All e.s.d.'s (except the e.s.d. in the dihedral angle between two l.s. planes) are estimated using the full covariance matrix. The cell e.s.d.'s are taken into account individually in the estimation of e.s.d.'s in distances, angles and torsion angles; correlations between e.s.d.'s in cell parameters are only used when they are defined by crystal symmetry. An approximate (isotropic) treatment of cell e.s.d.'s is used for estimating e.s.d.'s involving l.s. planes.
Refinement. Refinement of *F*^2^ against ALL reflections. The weighted *R*-factor *wR* and goodness of fit *S* are based on *F*^2^, conventional *R*-factors *R* are based on *F*, with *F* set to *z* 56*b*8 ero for negative *F*^2^. The threshold expression of *F*^2^ > σ(*F*^2^) is used only for calculating *R*-factors(gt) *etc*. and is not relevant to the choice of reflections for refinement. *R*-factors based on *F*^2^ are statistically about twice as large as those based on *F*, and *R*- factors based on ALL data will be even larger.

### Fractional atomic coordinates and isotropic or equivalent isotropic displacement parameters (Å^2^)

**Table:**

	*x*	*y*	*z*	*U*_iso_*/*U*_eq_	Occ. (<1)
Fe1	0.58172 (4)	0.31372 (3)	0.72928 (3)	0.03208 (11)	
Cl1	0.08364 (11)	0.27277 (9)	0.31279 (8)	0.0707 (3)	
O1W	−0.3468 (9)	−0.0077 (8)	0.1161 (7)	0.138 (2)	0.520 (5)
H1W1	−0.260 (3)	−0.007 (9)	0.100 (5)	0.208*	0.520 (5)
H1W2	−0.409 (6)	−0.071 (7)	0.064 (6)	0.208*	0.520 (5)
O1W'	−0.3403 (10)	0.1088 (9)	0.1280 (8)	0.138 (2)	0.480 (5)
H1W3	−0.434 (4)	0.075 (7)	0.089 (6)	0.208*	0.480 (5)
H1W4	−0.338 (13)	0.074 (5)	0.171 (4)	0.208*	0.480 (5)
O1A	0.6988 (7)	0.6549 (5)	0.9646 (4)	0.0645 (14)	0.622 (7)
O2A	0.8091 (7)	0.4809 (5)	1.0372 (4)	0.0649 (14)	0.622 (7)
O1B	0.6349 (13)	0.6377 (9)	0.9628 (8)	0.0645 (14)	0.378 (7)
O2B	0.7488 (13)	0.4655 (10)	1.0392 (8)	0.0649 (14)	0.378 (7)
O3	0.5582 (2)	0.1375 (2)	0.43325 (14)	0.0472 (5)	
O4	0.2819 (2)	0.02485 (19)	0.47266 (14)	0.0438 (5)	
O5	0.1944 (4)	0.3923 (3)	0.3485 (3)	0.1119 (15)	0.887 (4)
O6	0.0713 (5)	0.2023 (4)	0.2094 (2)	0.153 (2)	0.887 (4)
O7	−0.0680 (3)	0.2885 (4)	0.3206 (3)	0.1280 (16)	0.887 (4)
O8	0.1436 (6)	0.2088 (3)	0.3751 (3)	0.155 (2)	0.887 (4)
O5A	0.1129 (12)	0.3944 (5)	0.3903 (6)	0.1119 (15)	0.113 (4)
O6A	−0.0561 (7)	0.1927 (8)	0.3105 (8)	0.153 (2)	0.113 (4)
O7A	0.0631 (15)	0.2805 (10)	0.2154 (4)	0.1280 (16)	0.113 (4)
O8A	0.2168 (8)	0.2240 (9)	0.3367 (10)	0.155 (2)	0.113 (4)
N1	0.5132 (3)	0.4529 (2)	0.69872 (17)	0.0360 (5)	
N2	0.6593 (3)	0.4400 (2)	0.86762 (17)	0.0415 (6)	
N3	0.6736 (3)	0.2127 (2)	0.80085 (17)	0.0361 (5)	
N4	0.7769 (3)	0.3479 (2)	0.69072 (17)	0.0366 (5)	
N5	0.5024 (2)	0.1912 (2)	0.59060 (16)	0.0335 (5)	
N6	0.3654 (3)	0.2409 (2)	0.73032 (16)	0.0355 (5)	
C1	0.4350 (3)	0.4494 (3)	0.6055 (2)	0.0417 (6)	
H1	0.4102	0.3760	0.5466	0.050*	
C2	0.3910 (4)	0.5550 (3)	0.5964 (3)	0.0526 (8)	
H2	0.3361	0.5510	0.5317	0.063*	
C3	0.4283 (4)	0.6644 (3)	0.6823 (3)	0.0615 (9)	
H3	0.3998	0.7352	0.6766	0.074*	
C4	0.5083 (5)	0.6674 (3)	0.7770 (3)	0.0612 (9)	
H4	0.5347	0.7402	0.8367	0.073*	
C5	0.5489 (4)	0.5612 (3)	0.7822 (2)	0.0449 (7)	
C6	0.6359 (4)	0.5582 (3)	0.8833 (2)	0.0559 (9)	
C7	0.7348 (4)	0.4089 (3)	0.9468 (2)	0.0538 (8)	
C8	0.7429 (4)	0.2746 (3)	0.9043 (2)	0.0470 (7)	
C9	0.8163 (5)	0.2199 (4)	0.9657 (3)	0.0657 (10)	
H9	0.8666	0.2659	1.0363	0.079*	
C10	0.8141 (5)	0.0945 (4)	0.9200 (3)	0.0707 (10)	
H10	0.8604	0.0540	0.9599	0.085*	
C11	0.7430 (4)	0.0316 (3)	0.8159 (3)	0.0568 (8)	
H11	0.7417	−0.0523	0.7840	0.068*	
C12	0.6729 (3)	0.0915 (3)	0.7576 (2)	0.0424 (6)	
H12	0.6241	0.0470	0.6866	0.051*	
C13	0.9227 (3)	0.4269 (3)	0.7516 (2)	0.0467 (7)	
H13	0.9385	0.4723	0.8215	0.056*	
C14	1.0488 (4)	0.4418 (3)	0.7125 (3)	0.0570 (9)	
H14	1.1484	0.4963	0.7559	0.068*	
C15	1.0272 (4)	0.3770 (4)	0.6108 (3)	0.0609 (9)	
H15	1.1114	0.3880	0.5839	0.073*	
C16	0.8785 (3)	0.2938 (3)	0.5464 (3)	0.0492 (7)	
H16	0.8617	0.2477	0.4765	0.059*	
C17	0.7584 (3)	0.2822 (3)	0.5893 (2)	0.0373 (6)	
C18	0.5952 (3)	0.1934 (2)	0.5270 (2)	0.0356 (6)	
C19	0.3515 (3)	0.1088 (2)	0.55652 (19)	0.0330 (5)	
C20	0.2785 (3)	0.1384 (2)	0.64330 (19)	0.0345 (6)	
C21	0.1325 (3)	0.0665 (3)	0.6333 (2)	0.0464 (7)	
H21	0.0782	−0.0058	0.5739	0.056*	
C22	0.0673 (4)	0.1040 (4)	0.7139 (3)	0.0570 (9)	
H22	−0.0321	0.0577	0.7089	0.068*	
C23	0.1517 (4)	0.2100 (4)	0.8003 (3)	0.0559 (8)	
H23	0.1089	0.2379	0.8542	0.067*	
C24	0.3021 (3)	0.2759 (3)	0.8075 (2)	0.0429 (7)	
H24	0.3603	0.3464	0.8677	0.052*	

### Atomic displacement parameters (Å^2^)

**Table:**

	*U*^11^	*U*^22^	*U*^33^	*U*^12^	*U*^13^	*U*^23^
Fe1	0.03236 (19)	0.03226 (19)	0.02527 (17)	0.00582 (14)	0.00651 (14)	0.00684 (14)
Cl1	0.0706 (5)	0.0586 (5)	0.0855 (6)	0.0197 (4)	0.0324 (5)	0.0253 (5)
O1W	0.112 (4)	0.135 (4)	0.175 (6)	0.048 (4)	0.013 (4)	0.084 (5)
O1W'	0.112 (4)	0.135 (4)	0.175 (6)	0.048 (4)	0.013 (4)	0.084 (5)
O1A	0.088 (4)	0.0408 (16)	0.0411 (13)	0.015 (2)	0.003 (2)	0.0007 (12)
O2A	0.076 (4)	0.0571 (17)	0.0343 (12)	0.018 (2)	−0.009 (2)	0.0030 (12)
O1B	0.088 (4)	0.0408 (16)	0.0411 (13)	0.015 (2)	0.003 (2)	0.0007 (12)
O2B	0.076 (4)	0.0571 (17)	0.0343 (12)	0.018 (2)	−0.009 (2)	0.0030 (12)
O3	0.0503 (11)	0.0521 (12)	0.0297 (9)	0.0037 (9)	0.0138 (8)	0.0086 (9)
O4	0.0392 (10)	0.0434 (11)	0.0308 (9)	0.0001 (8)	0.0061 (8)	0.0017 (8)
O5	0.084 (2)	0.071 (2)	0.164 (4)	0.0011 (19)	0.025 (2)	0.045 (2)
O6	0.146 (4)	0.202 (5)	0.093 (3)	0.092 (4)	0.040 (3)	0.014 (3)
O7	0.089 (2)	0.135 (4)	0.187 (4)	0.047 (2)	0.084 (2)	0.061 (3)
O8	0.213 (5)	0.107 (3)	0.169 (5)	0.055 (3)	0.043 (4)	0.089 (3)
O5A	0.084 (2)	0.071 (2)	0.164 (4)	0.0011 (19)	0.025 (2)	0.045 (2)
O6A	0.146 (4)	0.202 (5)	0.093 (3)	0.092 (4)	0.040 (3)	0.014 (3)
O7A	0.089 (2)	0.135 (4)	0.187 (4)	0.047 (2)	0.084 (2)	0.061 (3)
O8A	0.213 (5)	0.107 (3)	0.169 (5)	0.055 (3)	0.043 (4)	0.089 (3)
N1	0.0355 (11)	0.0365 (11)	0.0351 (11)	0.0074 (9)	0.0103 (9)	0.0147 (9)
N2	0.0504 (13)	0.0337 (11)	0.0282 (10)	0.0090 (10)	0.0025 (10)	0.0052 (9)
N3	0.0358 (11)	0.0370 (11)	0.0333 (11)	0.0090 (9)	0.0107 (9)	0.0115 (9)
N4	0.0338 (10)	0.0342 (11)	0.0350 (11)	0.0042 (9)	0.0040 (9)	0.0125 (9)
N5	0.0301 (10)	0.0370 (11)	0.0265 (10)	0.0049 (9)	0.0088 (8)	0.0060 (9)
N6	0.0361 (11)	0.0378 (11)	0.0284 (10)	0.0075 (9)	0.0101 (8)	0.0092 (9)
C1	0.0352 (13)	0.0517 (16)	0.0361 (13)	0.0072 (12)	0.0089 (11)	0.0184 (12)
C2	0.0504 (16)	0.0660 (19)	0.0548 (17)	0.0216 (15)	0.0160 (13)	0.0375 (15)
C3	0.070 (2)	0.0612 (19)	0.075 (2)	0.0331 (16)	0.0305 (17)	0.0400 (17)
C4	0.083 (2)	0.0467 (17)	0.0556 (19)	0.0259 (17)	0.0209 (17)	0.0192 (15)
C5	0.0553 (16)	0.0360 (14)	0.0368 (14)	0.0100 (13)	0.0106 (12)	0.0103 (12)
C6	0.080 (2)	0.0402 (16)	0.0366 (15)	0.0173 (16)	0.0085 (15)	0.0087 (13)
C7	0.070 (2)	0.0501 (17)	0.0300 (14)	0.0170 (16)	0.0021 (13)	0.0107 (13)
C8	0.0546 (17)	0.0466 (16)	0.0360 (14)	0.0142 (14)	0.0079 (12)	0.0159 (12)
C9	0.086 (2)	0.069 (2)	0.0423 (17)	0.0322 (19)	0.0092 (16)	0.0248 (16)
C10	0.090 (2)	0.076 (2)	0.071 (2)	0.046 (2)	0.0302 (19)	0.0451 (19)
C11	0.0686 (19)	0.0473 (16)	0.069 (2)	0.0259 (15)	0.0326 (16)	0.0277 (15)
C12	0.0433 (14)	0.0413 (14)	0.0449 (15)	0.0119 (12)	0.0204 (12)	0.0153 (12)
C13	0.0346 (14)	0.0465 (16)	0.0463 (16)	0.0011 (12)	0.0009 (12)	0.0155 (13)
C14	0.0333 (14)	0.064 (2)	0.066 (2)	0.0034 (14)	0.0050 (14)	0.0280 (17)
C15	0.0365 (15)	0.077 (2)	0.075 (2)	0.0094 (15)	0.0228 (15)	0.0366 (19)
C16	0.0407 (14)	0.0607 (18)	0.0533 (16)	0.0136 (13)	0.0218 (12)	0.0261 (14)
C17	0.0361 (13)	0.0408 (13)	0.0377 (13)	0.0106 (11)	0.0131 (10)	0.0177 (11)
C18	0.0386 (13)	0.0373 (13)	0.0305 (12)	0.0100 (11)	0.0114 (10)	0.0125 (10)
C19	0.0328 (12)	0.0337 (12)	0.0271 (11)	0.0074 (10)	0.0054 (10)	0.0088 (10)
C20	0.0319 (12)	0.0367 (13)	0.0303 (12)	0.0081 (10)	0.0071 (10)	0.0100 (10)
C21	0.0363 (14)	0.0549 (17)	0.0410 (15)	0.0045 (13)	0.0113 (12)	0.0146 (13)
C22	0.0376 (15)	0.077 (2)	0.0498 (17)	0.0029 (15)	0.0177 (13)	0.0208 (16)
C23	0.0475 (16)	0.077 (2)	0.0464 (16)	0.0181 (15)	0.0254 (13)	0.0199 (16)
C24	0.0480 (15)	0.0450 (15)	0.0330 (13)	0.0125 (12)	0.0173 (11)	0.0091 (12)

### Geometric parameters (Å, °)

**Table:**

Fe1—N5	1.902 (2)	N6—C20	1.353 (3)
Fe1—N2	1.914 (2)	C1—C2	1.395 (4)
Fe1—N4	1.964 (2)	C1—H1	0.9300
Fe1—N3	1.974 (2)	C2—C3	1.371 (5)
Fe1—N6	1.976 (2)	C2—H2	0.9300
Fe1—N1	1.979 (2)	C3—C4	1.372 (5)
Cl1—O6	1.407 (3)	C3—H3	0.9300
Cl1—O7A	1.412 (4)	C4—C5	1.372 (4)
Cl1—O6A	1.414 (4)	C4—H4	0.9300
Cl1—O7	1.414 (3)	C5—C6	1.499 (4)
Cl1—O5A	1.417 (4)	C7—C8	1.503 (4)
Cl1—O8A	1.418 (4)	C8—C9	1.370 (5)
Cl1—O5	1.419 (3)	C9—C10	1.386 (5)
Cl1—O8	1.422 (3)	C9—H9	0.9300
O1W—H1W1	0.85 (2)	C10—C11	1.361 (5)
O1W—H1W2	0.84 (2)	C10—H10	0.9300
O1W—H1W3	1.43 (8)	C11—C12	1.375 (4)
O1W—H1W4	0.99 (7)	C11—H11	0.9300
O1W'—H1W3	0.828 (19)	C12—H12	0.9300
O1W'—H1W4	0.85 (2)	C13—C14	1.381 (5)
O1A—C6	1.246 (5)	C13—H13	0.9300
O2A—C7	1.230 (6)	C14—C15	1.354 (5)
O1B—C6	1.211 (10)	C14—H14	0.9300
O2B—C7	1.237 (11)	C15—C16	1.394 (4)
O3—C18	1.220 (3)	C15—H15	0.9300
O4—C19	1.206 (3)	C16—C17	1.365 (4)
N1—C5	1.346 (3)	C16—H16	0.9300
N1—C1	1.347 (3)	C17—C18	1.499 (4)
N2—C7	1.378 (4)	C19—C20	1.508 (4)
N2—C6	1.385 (4)	C20—C21	1.372 (4)
N3—C12	1.342 (4)	C21—C22	1.392 (4)
N3—C8	1.350 (4)	C21—H21	0.9300
N4—C13	1.351 (3)	C22—C23	1.364 (5)
N4—C17	1.358 (3)	C22—H22	0.9300
N5—C18	1.375 (3)	C23—C24	1.391 (4)
N5—C19	1.383 (3)	C23—H23	0.9300
N6—C24	1.337 (3)	C24—H24	0.9300
			
N5—Fe1—N2	178.54 (10)	C2—C3—C4	118.6 (3)
N5—Fe1—N4	82.29 (9)	C2—C3—H3	120.7
N2—Fe1—N4	97.79 (10)	C4—C3—H3	120.7
N5—Fe1—N3	99.81 (9)	C3—C4—C5	118.9 (3)
N2—Fe1—N3	81.65 (9)	C3—C4—H4	120.6
N4—Fe1—N3	90.49 (9)	C5—C4—H4	120.6
N5—Fe1—N6	82.06 (9)	N1—C5—C4	123.3 (3)
N2—Fe1—N6	97.90 (10)	N1—C5—C6	115.2 (3)
N4—Fe1—N6	164.25 (9)	C4—C5—C6	121.5 (3)
N3—Fe1—N6	90.42 (9)	O1B—C6—N2	127.9 (6)
N5—Fe1—N1	96.77 (9)	O1A—C6—N2	126.1 (4)
N2—Fe1—N1	81.76 (10)	O1B—C6—C5	118.5 (6)
N4—Fe1—N1	91.62 (9)	O1A—C6—C5	122.9 (4)
N3—Fe1—N1	163.41 (9)	N2—C6—C5	110.1 (2)
N6—Fe1—N1	91.98 (9)	O2A—C7—N2	127.3 (4)
O6—Cl1—O6A	94.3 (5)	O2B—C7—N2	124.6 (6)
O7A—Cl1—O6A	110.0 (4)	O2A—C7—C8	121.7 (4)
O6—Cl1—O7	110.5 (2)	O2B—C7—C8	121.9 (6)
O7A—Cl1—O7	96.5 (6)	N2—C7—C8	110.1 (2)
O6A—Cl1—O7	46.1 (4)	N3—C8—C9	122.7 (3)
O6—Cl1—O5A	146.3 (5)	N3—C8—C7	114.7 (3)
O7A—Cl1—O5A	109.7 (4)	C9—C8—C7	122.5 (3)
O6A—Cl1—O5A	109.4 (4)	C8—C9—C10	118.5 (3)
O7—Cl1—O5A	73.5 (4)	C8—C9—H9	120.7
O6—Cl1—O8A	84.0 (6)	C10—C9—H9	120.7
O7A—Cl1—O8A	109.5 (4)	C11—C10—C9	118.8 (3)
O6A—Cl1—O8A	109.3 (4)	C11—C10—H10	120.6
O7—Cl1—O8A	150.4 (5)	C9—C10—H10	120.6
O5A—Cl1—O8A	109.0 (4)	C10—C11—C12	120.4 (3)
O6—Cl1—O5	109.3 (2)	C10—C11—H11	119.8
O7A—Cl1—O5	82.8 (4)	C12—C11—H11	119.8
O6A—Cl1—O5	152.3 (4)	N3—C12—C11	121.4 (3)
O7—Cl1—O5	109.7 (2)	N3—C12—H12	119.3
O8A—Cl1—O5	87.9 (4)	C11—C12—H12	119.3
O6—Cl1—O8	108.8 (2)	N4—C13—C14	121.4 (3)
O7A—Cl1—O8	144.5 (5)	N4—C13—H13	119.3
O6A—Cl1—O8	75.9 (4)	C14—C13—H13	119.3
O7—Cl1—O8	110.1 (2)	C15—C14—C13	119.9 (3)
O5A—Cl1—O8	100.3 (4)	C15—C14—H14	120.0
O5—Cl1—O8	108.3 (2)	C13—C14—H14	120.0
H1W1—O1W—H1W2	99 (3)	C14—C15—C16	119.8 (3)
H1W1—O1W—H1W3	115 (9)	C14—C15—H15	120.1
H1W2—O1W—H1W3	92 (8)	C16—C15—H15	120.1
H1W1—O1W—H1W4	111 (8)	C17—C16—C15	117.8 (3)
H1W2—O1W—H1W4	145 (8)	C17—C16—H16	121.1
H1W3—O1W—H1W4	61 (4)	C15—C16—H16	121.1
H1W3—O1W'—H1W4	100 (3)	N4—C17—C16	123.3 (3)
C5—N1—C1	118.1 (3)	N4—C17—C18	115.0 (2)
C5—N1—Fe1	114.70 (19)	C16—C17—C18	121.8 (3)
C1—N1—Fe1	127.2 (2)	O3—C18—N5	128.6 (2)
C7—N2—C6	123.1 (2)	O3—C18—C17	121.5 (2)
C7—N2—Fe1	118.6 (2)	N5—C18—C17	109.9 (2)
C6—N2—Fe1	118.30 (19)	O4—C19—N5	128.7 (3)
C12—N3—C8	118.1 (3)	O4—C19—C20	122.2 (2)
C12—N3—Fe1	126.92 (19)	N5—C19—C20	109.0 (2)
C8—N3—Fe1	114.95 (19)	N6—C20—C21	122.7 (3)
C13—N4—C17	117.8 (2)	N6—C20—C19	115.6 (2)
C13—N4—Fe1	128.3 (2)	C21—C20—C19	121.7 (2)
C17—N4—Fe1	113.95 (17)	C20—C21—C22	118.7 (3)
C18—N5—C19	122.9 (2)	C20—C21—H21	120.6
C18—N5—Fe1	117.98 (16)	C22—C21—H21	120.6
C19—N5—Fe1	119.04 (17)	C23—C22—C21	118.8 (3)
C24—N6—C20	118.1 (2)	C23—C22—H22	120.6
C24—N6—Fe1	127.95 (19)	C21—C22—H22	120.6
C20—N6—Fe1	113.76 (17)	C22—C23—C24	119.7 (3)
N1—C1—C2	120.7 (3)	C22—C23—H23	120.1
N1—C1—H1	119.7	C24—C23—H23	120.1
C2—C1—H1	119.7	N6—C24—C23	121.8 (3)
C3—C2—C1	120.4 (3)	N6—C24—H24	119.1
C3—C2—H2	119.8	C23—C24—H24	119.1
C1—C2—H2	119.8		
			
N5—Fe1—N1—C5	179.5 (2)	Fe1—N2—C6—O1A	−168.7 (5)
N2—Fe1—N1—C5	−0.5 (2)	C7—N2—C6—C5	−179.3 (3)
N4—Fe1—N1—C5	−98.1 (2)	Fe1—N2—C6—C5	0.3 (4)
N3—Fe1—N1—C5	−0.9 (4)	N1—C5—C6—O1B	−160.9 (7)
N6—Fe1—N1—C5	97.2 (2)	C4—C5—C6—O1B	18.5 (8)
N5—Fe1—N1—C1	0.3 (2)	N1—C5—C6—O1A	168.7 (4)
N2—Fe1—N1—C1	−179.7 (2)	C4—C5—C6—O1A	−11.9 (7)
N4—Fe1—N1—C1	82.7 (2)	N1—C5—C6—N2	−0.7 (4)
N3—Fe1—N1—C1	179.9 (3)	C4—C5—C6—N2	178.8 (3)
N6—Fe1—N1—C1	−82.0 (2)	C6—N2—C7—O2A	−10.2 (7)
N4—Fe1—N2—C7	−89.8 (3)	Fe1—N2—C7—O2A	170.2 (5)
N3—Fe1—N2—C7	−0.4 (2)	C6—N2—C7—O2B	21.4 (8)
N6—Fe1—N2—C7	88.8 (3)	Fe1—N2—C7—O2B	−158.2 (7)
N1—Fe1—N2—C7	179.7 (3)	C6—N2—C7—C8	−179.3 (3)
N4—Fe1—N2—C6	90.6 (3)	Fe1—N2—C7—C8	1.1 (4)
N3—Fe1—N2—C6	180.0 (3)	C12—N3—C8—C9	2.1 (5)
N6—Fe1—N2—C6	−90.8 (3)	Fe1—N3—C8—C9	−178.2 (3)
N1—Fe1—N2—C6	0.1 (2)	C12—N3—C8—C7	−178.6 (3)
N5—Fe1—N3—C12	−0.7 (2)	Fe1—N3—C8—C7	1.2 (4)
N2—Fe1—N3—C12	179.3 (2)	O2A—C7—C8—N3	−171.2 (4)
N4—Fe1—N3—C12	−82.9 (2)	O2B—C7—C8—N3	158.5 (6)
N6—Fe1—N3—C12	81.3 (2)	N2—C7—C8—N3	−1.4 (4)
N1—Fe1—N3—C12	179.7 (3)	O2A—C7—C8—C9	8.1 (7)
N5—Fe1—N3—C8	179.6 (2)	O2B—C7—C8—C9	−22.1 (8)
N2—Fe1—N3—C8	−0.5 (2)	N2—C7—C8—C9	177.9 (3)
N4—Fe1—N3—C8	97.3 (2)	N3—C8—C9—C10	−2.4 (6)
N6—Fe1—N3—C8	−98.4 (2)	C7—C8—C9—C10	178.3 (4)
N1—Fe1—N3—C8	0.0 (4)	C8—C9—C10—C11	1.7 (6)
N5—Fe1—N4—C13	−174.4 (3)	C9—C10—C11—C12	−0.8 (6)
N2—Fe1—N4—C13	7.1 (3)	C8—N3—C12—C11	−1.1 (4)
N3—Fe1—N4—C13	−74.6 (3)	Fe1—N3—C12—C11	179.2 (2)
N6—Fe1—N4—C13	−167.9 (3)	C10—C11—C12—N3	0.5 (5)
N1—Fe1—N4—C13	89.0 (3)	C17—N4—C13—C14	0.6 (4)
N5—Fe1—N4—C17	5.74 (19)	Fe1—N4—C13—C14	−179.3 (2)
N2—Fe1—N4—C17	−172.78 (19)	N4—C13—C14—C15	0.5 (5)
N3—Fe1—N4—C17	105.6 (2)	C13—C14—C15—C16	−1.1 (6)
N6—Fe1—N4—C17	12.3 (5)	C14—C15—C16—C17	0.7 (5)
N1—Fe1—N4—C17	−90.9 (2)	C13—N4—C17—C16	−1.0 (4)
N4—Fe1—N5—C18	−9.1 (2)	Fe1—N4—C17—C16	178.9 (2)
N3—Fe1—N5—C18	−98.3 (2)	C13—N4—C17—C18	178.2 (2)
N6—Fe1—N5—C18	172.7 (2)	Fe1—N4—C17—C18	−1.9 (3)
N1—Fe1—N5—C18	81.6 (2)	C15—C16—C17—N4	0.4 (5)
N4—Fe1—N5—C19	174.3 (2)	C15—C16—C17—C18	−178.8 (3)
N3—Fe1—N5—C19	85.2 (2)	C19—N5—C18—O3	8.5 (5)
N6—Fe1—N5—C19	−3.9 (2)	Fe1—N5—C18—O3	−167.9 (2)
N1—Fe1—N5—C19	−94.9 (2)	C19—N5—C18—C17	−173.5 (2)
N5—Fe1—N6—C24	−178.0 (3)	Fe1—N5—C18—C17	10.0 (3)
N2—Fe1—N6—C24	0.5 (3)	N4—C17—C18—O3	173.2 (3)
N4—Fe1—N6—C24	175.4 (3)	C16—C17—C18—O3	−7.6 (4)
N3—Fe1—N6—C24	82.1 (3)	N4—C17—C18—N5	−4.9 (3)
N1—Fe1—N6—C24	−81.4 (2)	C16—C17—C18—N5	174.3 (3)
N5—Fe1—N6—C20	6.44 (19)	C18—N5—C19—O4	4.4 (5)
N2—Fe1—N6—C20	−175.04 (19)	Fe1—N5—C19—O4	−179.2 (2)
N4—Fe1—N6—C20	−0.1 (5)	C18—N5—C19—C20	−175.6 (2)
N3—Fe1—N6—C20	−93.42 (19)	Fe1—N5—C19—C20	0.8 (3)
N1—Fe1—N6—C20	103.00 (19)	C24—N6—C20—C21	−3.0 (4)
C5—N1—C1—C2	−0.7 (4)	Fe1—N6—C20—C21	173.0 (2)
Fe1—N1—C1—C2	178.4 (2)	C24—N6—C20—C19	176.1 (2)
N1—C1—C2—C3	0.7 (5)	Fe1—N6—C20—C19	−7.8 (3)
C1—C2—C3—C4	−0.5 (5)	O4—C19—C20—N6	−175.3 (2)
C2—C3—C4—C5	0.3 (6)	N5—C19—C20—N6	4.7 (3)
C1—N1—C5—C4	0.6 (5)	O4—C19—C20—C21	3.9 (4)
Fe1—N1—C5—C4	−178.6 (3)	N5—C19—C20—C21	−176.1 (3)
C1—N1—C5—C6	−180.0 (3)	N6—C20—C21—C22	3.3 (5)
Fe1—N1—C5—C6	0.8 (4)	C19—C20—C21—C22	−175.8 (3)
C3—C4—C5—N1	−0.4 (5)	C20—C21—C22—C23	−0.8 (5)
C3—C4—C5—C6	−179.8 (3)	C21—C22—C23—C24	−1.8 (5)
C7—N2—C6—O1B	−21.4 (9)	C20—N6—C24—C23	0.3 (4)
Fe1—N2—C6—O1B	158.2 (7)	Fe1—N6—C24—C23	−175.1 (2)
C7—N2—C6—O1A	11.7 (7)	C22—C23—C24—N6	2.1 (5)

### Hydrogen-bond geometry (Å, °)

**Table:**

*D*—H···*A*	*D*—H	H···*A*	*D*···*A*	*D*—H···*A*
C12—H12···O3^i^	0.93	2.30	3.084 (3)	142.
C12—H12···O4^i^	0.93	2.55	3.288 (4)	136.
C2—H2···O5	0.93	2.52	3.292 (5)	140.
C2—H2···O5A	0.93	2.41	3.116 (8)	133.
C11—H11···O6^i^	0.93	2.62	3.385 (6)	140.
C11—H11···O8A^i^	0.93	2.32	3.191 (11)	156.
C16—H16···O6A^ii^	0.93	2.58	3.431 (10)	153.

Symmetry codes: (i) −*x*+1, −*y*, −*z*+1; (ii) *x*+1, *y*, *z*.
